# Macrophage Phenotypes Regulate Scar Formation and Chronic Wound Healing

**DOI:** 10.3390/ijms18071545

**Published:** 2017-07-17

**Authors:** Mark Hesketh, Katherine B. Sahin, Zoe E. West, Rachael Z. Murray

**Affiliations:** The Institute for Health and Biomedical Innovation, School of Biomedical Sciences, Faculty of Health, Queensland University of Technology, Brisbane QLD 4059, Australia; m.hesketh@connect.qut.edu.au (M.H.); katherine.sahin@connect.qut.edu.au (K.B.S.); zoe.west@connect.qut.edu.au (Z.E.W.)

**Keywords:** macrophage, monocyte, wound healing, fibrosis, chronic wound, diabetes, chronic venous disease

## Abstract

Macrophages and inflammation play a beneficial role during wound repair with macrophages regulating a wide range of processes, such as removal of dead cells, debris and pathogens, through to extracellular matrix deposition re-vascularisation and wound re-epithelialisation. To perform this range of functions, these cells develop distinct phenotypes over the course of wound healing. They can present with a pro-inflammatory M1 phenotype, more often found in the early stages of repair, through to anti-inflammatory M2 phenotypes that are pro-repair in the latter stages of wound healing. There is a continuum of phenotypes between these ranges with some cells sharing phenotypes of both M1 and M2 macrophages. One of the less pleasant consequences of quick closure, namely the replacement with scar tissue, is also regulated by macrophages, through their promotion of fibroblast proliferation, myofibroblast differentiation and collagen deposition. Alterations in macrophage number and phenotype disrupt this process and can dictate the level of scar formation. It is also clear that dysregulated inflammation and altered macrophage phenotypes are responsible for hindering closure of chronic wounds. The review will discuss our current knowledge of macrophage phenotype on the repair process and how alterations in the phenotypes might alter wound closure and the final repair quality.

## 1. Introduction

To overcome the repeated dermal insults and injuries that occur on a daily basis, evolution has provided a swift but robust mechanism for tissue repair. The drawback of this hasty mechanism is scar formation and imbalances in this mechanism can lead to non-healing wounds. The repair process is highly complex, consisting of four stages: haemostasis, followed by inflammatory, proliferative and remodeling phases, that involve complex interactions between skin cells, immune cells and extracellular matrix (ECM) components, as well as a plethora of soluble mediators that help orchestrate the process [1,2]. At the start of the repair process, blood loss is stemmed by formation of a blood clot containing platelets, red blood cells, white blood cells and fibrin fibers. These fibers then act as a scaffold for immune cells that are attracted by soluble factors released from platelets and injured tissue, to enter the wound. Mouse wound models have shown that within hours neutrophils enter the injured skin along with a small wave of pioneer monocytes that leak into tissue through areas of microhemorrhaging [3,4]. Neutrophil numbers peak around days 1–2 [4]. These cells are the first responders and kill potential pathogens, debride the wound, and release a number of soluble mediators that attract other immune cells to the site of injury. Once they have undergone their role in the repair process, they apoptose. Monocytes begin migrating into the wound and en route they differentiate into wound associated macrophages in a process driven by factors in the extracellular milieu [2]. Wound associated macrophage numbers then increase until day 2 when their numbers remain stable until around day 5 when they begin to gradually decrease to steady state levels by day 14 [5]. During this time in the wound, macrophages remove spent neutrophils and secrete various cytokines, growth factors and other mediators [6]. Through these mediators, macrophages either directly or indirectly regulate the proliferative stage. They stimulate fibroblasts, keratinocytes and endothelial cells to differentiate, proliferate and migrate, leading to the deposition of new ECM, re-epithelialisation and neovascularisation of the wound [7]. In the remodelling phase, macrophages can release enzymes that alter the composition of the ECM and the structure of the wound.

## 2. Macrophages Phenotypes

**Figure 1:**
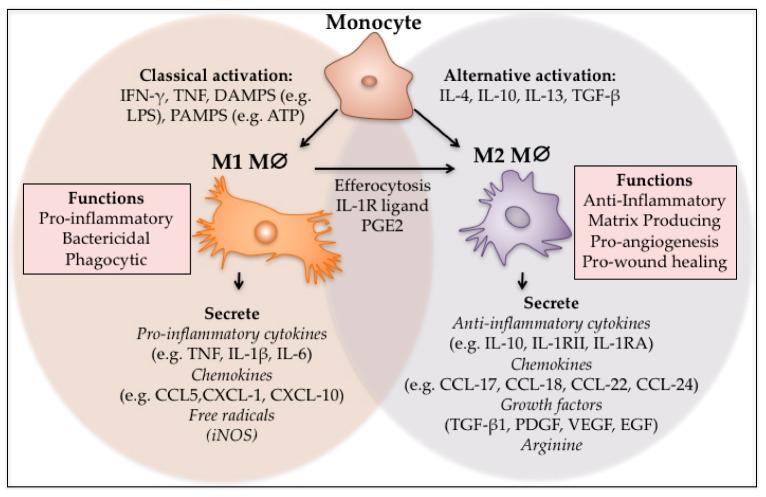
M1 and M2 polarisation of macrophages. Monocytes can be classically or alternatively activated to form M1 and M2 macrophages respectively. M1 macrophages can also differentiate into M2 macrophages through local cues and after efferocytosis. The M1 phenotype is pro-inflammatory, phagocytic and bactericidal, while the M2 macrophages act to switch off inflammation and regulate re-vascularisation and wound closure.

Differentiated wound macrophages are not a homogeneous population of cells but exist as multiple phenotypes that can be broadly classified as M1 and M2 phenotypes (Table 1) [8,9,10]. These are not discrete populations in vivo but instead they represent a continuum of phenotypes from M1-M2 that evolve as the wound matures. Pro-inflammatory mediators interferon-γ (IFN-γ) and tumour necrosis factor (TNF), and damage associated pattern molecules (DAMPs) activate cells to produce ‘classically activated’ M1 macrophages (Figure 1) [11]. This results in a pro-inflammatory macrophage phenotype that prolifically produces pro-inflammatory cytokines, such as TNF and Interleukin-6 (IL-6), and other mediators that facilitate the initial stages of wound healing (Figure 1). These M1 cells are highly phagocytic, enabling them to phagocytose neutrophils that have apoptosed and remove any pathogens or debris in the wound. Alternatively activated M2 macrophages are typically anti-inflammatory and can be produced in vitro through the addition of IL-4 or IL-13 or through their phagocytosis of apoptosed neutrophils. M2 cells have been divided into four discrete types—M2a, M2b, M2c and M2d—in vitro based on their function and key markers (Table 1) [12]. Whether they all exist in wounds is currently unclear. There is some controversy in the literature as to how the M2 wound phenotype is derived [13]. Different wound macrophage subsets could be derived from monocytes with different phenotypes recruited at different times that differentiate into macrophages with distinct phenotypes, or monocytes could be recruited at different times in the course of wound healing where the ever-changing wound milieu affecting their polarisation, or M1 macrophages could differentiate into M2 macrophages driven by cues in the local wound environment [13]. A number of studies point to the latter suggestion that it is the same macrophages that regulate early inflammatory functions and later wound reparative functions [13,14]. This suggests that the local environment or some function of the macrophage results in their switch in phenotype in wounds.

## 3. Macrophages Regulate Wound Closure and Scar Formation

Historically, a number of studies addressing the role of macrophages in wound healing provided some doubt as to the necessity and role of these cells in the repair process [22,23]. Depletion of macrophages using anti-serum suggested that macrophages were necessary for the repair process [22]. However, these studies required steroids to deplete most macrophages and so this may also have affected the repair process. The advent of genetic engineering mice has since improved our understanding of the role in macrophages in repair. Mice that lack macrophages (PU.1 mice) repair skin at the same rate as control mice with greatly reduced scar formation, suggesting that macrophages regulate scar formation but not wound closure [23]. However, the PU.1 mice also lack functioning neutrophils [23]. More recently, elegant studies using diphtheria toxin-driven lysozyme M-specific ablation or CD11b specific cell lineage ablation to selectively deplete all macrophage phenotypes, show that these cells are necessary for timely wound healing and they regulate scar formation [24,25,26]. Depletion of macrophages using the diphtheria toxin driven (DTR) lysozyme M-specific strategy just prior to and throughout the repair process leads to impaired wound healing compared with control mice [24]. Without macrophages, wounds are not cleared of neutrophils and contain high levels of pro-inflammatory cytokines [24]. They contain less anti-inflammatory cytokines, so the ability to dampen inflammation in the wound is reduced, as is the expression of transforming growth factor-beta 1 (TGF-β1), a growth factor that has roles in regulating proliferation, migration, differentiation, and ECM production during the repair process [24]. Consequently, reduced myofibroblast differentiation leads to less wound contraction, and a temporal shift in VEGF expression to later time points resulting in a reduction in angiogenesis [24]. Similar results were seen when ablating CD11b macrophages with diphtheria toxin, except, curiously, there were no changes in wound neutrophil levels [25]. These results suggest that macrophages are necessary for efficient repair.

Wound macrophages are not a homogenous population of cells and at different stages of the repair process they can have distinct phenotypes. Using the diphtheria toxin driven lysozyme M-specific strategy, macrophages have been selectively depleted at distinct times in the repair process to tease out the roles of these macrophages over the course of wound healing [26]. While loss of macrophages during the final stages of repair had no impact on tissue maturation, the removal of macrophages in the mid repair stage resulted in wounds that were significantly delayed with signs of haemorrhaging, suggesting they play a crucial role at this point of the repair process. The mid repair macrophages, presumably M2 macrophages, were found to be required for vascular stability through their production of vascular epithelial growth factor-A (VEGF-A) and TGF-β1 [26]. Depletion of macrophages only in the early stages (days 1–5) suggests macrophages regulate the degree of scar formation and that this pool might be a good target for reducing scar formation [26]. Loss of the early pool of macrophages did significantly delay wound closure, but macrophage repopulation of the wound rescued this delay with wounds reepithelialised by day 14 similar to the control mice [26]. Attractively, these latter wounds have reduced fibrosis, indicating that this early pool of macrophages is necessary for scar formation [26]. This suggests that having less macrophages in the early stages of the repair process might be beneficial in terms of reducing scar formation. This data together suggests that macrophages regulate wound closure and scar formation, and macrophages at different stages of the repair process perform different functions. Interestingly, the distinct roles of different macrophage populations in fibrosis and on the repair process is not limited to skin repair [27]. In liver injury, macrophage depletion using a CD11b-DTR macrophage mouse model shows that during the progressive inflammatory liver injury stage a lack of macrophages reduces fibrosis [27]. However, macrophage depletion during the recovery phase leads to failure of resolution with persistence of cellular and matrix components of the fibrotic response [27]. This suggests that macrophages can also play dual roles during the repair process in other types of injury.

While macrophages found in the wound are derived predominantly from circulatory monocytes, skin does contain low numbers of resident macrophages [8]. The exact contribution of these resident macrophages during the repair process is currently unclear. They potentially play a role in the initial recruitment of immune cells to the site of injury, although the absence of skin macrophages prior to wounding does not affect the overall recruitment of neutrophils [24]. It is known that alternatively activating macrophages derived from monocytes and from tissue macrophages can produce phenotypically and functionally distinct macrophages [28]. This suggests that the small population of resident macrophages might play different roles to the recruited macrophages derived from monocytes during the wound repair process, but this has yet to be tested.

## 4. M2 Macrophages and Wound Closure

Wounds contain macrophages with phenotypes associated with both classical and alternative activation, the ratio of which alters as the wound matures [29]. During the early stages of inflammation, around 85% of macrophages have an M1 phenotype and avidly secrete pro-inflammatory mediators, while 15% have an M2 phenotype that secrete anti-inflammatory cytokines and growth factors [29]. This ratio switches as the wound matures so that 5–7 days post injury only 15–20% of macrophages have an M1 phenotype and the wound is primarily populated by cells with an M2 phenotype [29,30]. At 5 days post injury, M2 macrophages dominating the acute wound express CD301b, along with high levels of the anti-inflammatory cytokine IL-10 and growth factors such as platelet-derived growth factor (PDGFβ), and TGF-β1 [30]. By using mice expressing a diphtheria toxin receptor/GFP fusion protein under the endogenous CD301b promoter to deplete these CD301b expressing population of M2 cells in mice wounds 3 days post injury, it has been shown that these cells are necessary for timely re-epithelisation, revascularization and fibroblast regeneration [30]. This mirrors the results seen when both M1 and M2 phenotypes are depleted [24,25,26]. To further tease out the role of the M1 and M2 phenotypes, the cFMS kinase inhibitor GW2580 has been used to selectively inhibit the transition from M1 to M2 macrophages in an acute wound [31]. Blocking this transition prolongs the inflammatory phase; mice treated with this inhibitor prior to and during wound healing have higher number of neutrophils and macrophages at 14 days post injury compared to untreated mice [31]. M2 macrophages secrete factors, such as TGF-β1, that induce the proliferation of fibroblasts and their differentiation into myofibroblasts. These cells are responsible for collagen production in the wound. Consequently, the GW2580 treated mice have significantly reduced levels of total collagen suggesting that M2 macrophages regulate scar formation.

Since M2 cells have been found to promote repair, it has been proposed that increasing M2 macrophage numbers in the wound might accelerate wound closure. Transplantation of the CD301b M2 population from a day 5 wound into a day 3 wound increases fibroblast proliferation and vascular regeneration, confirming that these CD301b positive M2 macrophages are key cells regulating the reparative phases during wound healing [30]. They do not, however, alter wound closure rates [30]. Similarly, injection of anti-inflammatory M2a or M2c macrophages, that have been stimulated in vitro with IL-4 or IL-10 respectively, into excisional wounds of mice had no effect on wound closure [32]. However, data from chronic diabetic wounds suggest that it may well be how the M2 macrophage is activated that is crucial for timely wound healing, and that IL-4 or IL-10 are not key drivers of the reparative M2 phenotype in wounds. Collectively, these results suggest that in an acute wound the extended and/or increased presence of M1 macrophages, reduced numbers of M2 macrophages and/or increased M2 activation potentially dictate repair and the level of scar formation in part through their secretion of TGF-β1. High levels of TGF-β1 lead to an expansion of fibroblast population and increased differentiation into myofibroblasts, which then secrete more collagen, strengthening the wound. However, excess collagen increases fibrosis. Collectively, these results suggest that M2 macrophages regulate re-vascularisation, fibroblast regeneration and myofibroblast differentiation and collagen production.

## 5. Macrophage Phenotype and Dysregulated Inflammation in Chronic Wounds

### 5.1. Chronic Wound Macrophages Are Predominantly M1 Pro-Inflammatory Cells

**Figure 2:**
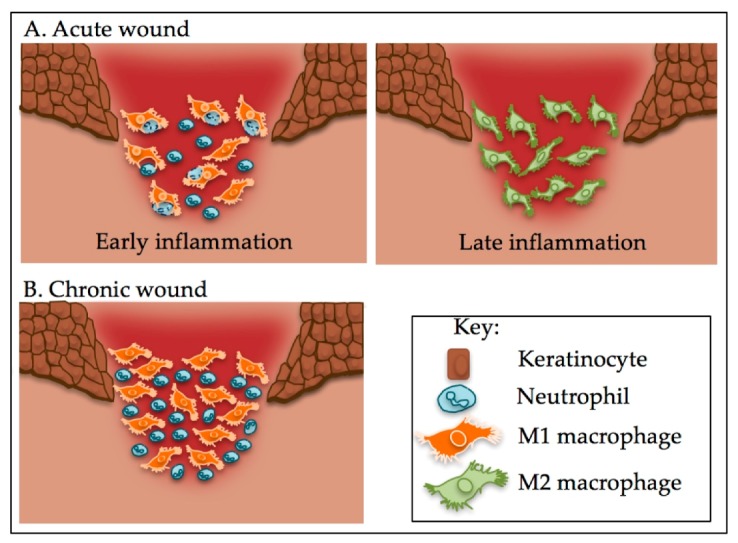
The M1 to M2 switch is dysregulated in chronic wounds and, unlike in acute wounds, macrophages are unable to phagocytose neutrophils. (**A**) In the early inflammatory stage of an acute wound, macrophages phagocytose spent neutrophils. In the later stages, having performed this role, macrophages switch phenotype and are predominantly M2 macrophages; (**B**) In a chronic wound, macrophages are predominantly M1 that are unable phagocytosis spent neutrophils. This leads to the recruitment of more macrophages and an increase in inflammation.

Chronic wounds, such as diabetic foot ulcers, venous leg ulcers, and pressure ulcers, do not heal in a timely manner. The pathophysiology of these chronic wounds is complex and can include diverse factors such diabetic status, venous insufficiency, arterial perfusion or persistent pressure [33]. What these wounds all have in common is an increased and prolonged inflammatory stage with a distinct wound cell composition, showing changes in both in cell number and the predominant macrophage phenotype, compared to acute wounds [34,35,36]. The typical shift in M1 to M2 macrophages seen in acute wounds is dysregulated in chronic wounds (Figure 2) [34,35,36,37]. At the chronic wound margin, approximately 80% of cells are pro-inflammatory M1 macrophages with these cells playing a major role in the pathogenesis and the on-going chronicity of wounds [35]. Mouse models of diabetic wounds show that reduced M2 macrophage levels lead to a reduction in growth factor levels that regulate the proliferative stage of repair, such as TGFβ1, insulin-like growth factor-1 (IGF-1) and vascular endothelial growth factor (VEGF) [37,38]. These wounds contain high levels of pro-inflammatory cytokines and mediators, such as TNF, IL-1β, IL-17 and iNOS, which contribute to the non-healing phenotype [5,35,37,38,39]. Patients with chronic wounds present with high levels of pro-inflammatory cytokines in their wound fluid [35,40,41]. At a cellular level, high levels of pro-inflammatory mediators, such as TNF in the wound extracellular milieu, lead to alterations in the secretion of MMP-1, MMP-3 and TIMP-1 from dermal fibroblasts [42]. This changes the fine balance between MMPs and TIMPs, leading to the detrimental excessive ECM proteolysis seen in the wound environment, which contributes to wound chronicity [42].

### 5.2. Hyperglycaemia Alters Efferocytosis and the Macrophage Phenotypic Switch in Diabetes

Alternative activation and macrophage phenotype can be driven by factors in the extracellular environment. In vitro M2 alternate activation is driven by stimulation with IL-4/IL-13, but in an acute wound extracellular IL-4 and IL-13 is lacking [29]. This, along with the fact that transplantation of in vitro IL-4 or IL-10 differentiated M2 macrophages has no effect on wound closure, suggest that there may be some other driver of differentiation and perhaps a different phenotype involved [32]. Other than factors in the local environment, macrophage phenotype is also altered after the phagocytosis of neutrophils, in a process known as efferocytosis (‘to carry to the grave’) [43,44,45]. In vitro, macrophages that engulf apoptosed neutrophils switch to an M2b phenotype that expresses low levels of IL-12 and produces high levels of IL-10 and TGF-β [46]. During the initial stages of wound healing, neutrophils enter the wound and apoptose once they have performed their roles in clearing pathogens and debris. These spent neutrophils are removed by macrophages, which in turn is proposed to lead to their switching to an anti-inflammatory M2 phenotype that dampens inflammation and so aids the resolution of wounds. In acute wounds, neutrophil efferocytosis drives the resolution of inflammation through the induction of micro-RNA-21, which silences the pro-inflammatory activity of the programmed cell death 4 (*PDCD4*) gene and favours cJun-AP1 activity leading to an increased production of IL-10, a major suppressor of inflammation [47]. Thus, the efferocytosis of neutrophils, and possibly the local environment, can determine whether macrophages differentiate and effectively resolve inflammation in a timely manner [43]. Alterations in external cues and in efferocytosis could prevent these macrophages from differentiating, leaving a pro-inflammatory phenotype that amplifies inflammation and contributes to the impaired wound resolution phenotype.

Monocytes can be preprogramed prior to entering the wound environment, particularly in diseases that induce a chronic state of inflammation. In diabetes, the diabetic environment triggers changes in haematopoietic cells generating monocytes that are pre-programmed towards a pro-inflammatory phenotype. Hyperglycemia and the associated advanced glycated end products (AGEs) alter macrophages such that they present an impaired ability to efferocytose spent neutrophils [48,49,50,51]. This results in high levels of neutrophils in diabetic wounds. In mice diabetic models, the increase in apoptotic cells leads to increases in pro-inflammatory cytokines TNF and IL-6 in tissue and wound fluid, with a concomitant decrease in the anti-inflammatory cytokine IL-10 that would normally act to turn off the inflammatory response [48,49]. TNF is one of the key cytokines that can impair wound healing [52]. Increased apoptotic cell load leads to increased inflammation as seen in chronic wounds. Increased apoptosis in an acute wound model leads to an increased inflammatory response with reduced anti-inflammatory cytokines [48]. This, along with the lack of IL-4 and IL-13 in acute wounds suggests that engulfment of apoptotic cells is a major trigger pushing macrophages from an M1 pro-inflammatory phenotype towards the M2 anti-inflammatory phenotype to resolve inflammation (Figure 2).

### 5.3. Iron Regulates the Macrophage Phenotype in Chronic Venous Ulcers (CVU)

Like diabetes, chronic venous disease is a chronic inflammatory disease. These patients have persistently raised venous pressure (venous hypertension) in lower limb deep and superficial veins. Increased pressure and shear stress leads to changes in the endothelium and the activation of leukocytes including monocytes, macrophages and neutrophils which become “trapped” in the limbs. These activated leukocytes in turn cause injury to the endothelium, leading to a chronic inflammatory state. So, like the diabetic monocytes/macrophages, these cells also have an altered phenotype prior to entering a wound. Within a human chronic venous ulcer (CVU), approximately 80% of the cells at wound margin are macrophages, similar to what is seen in diabetic wounds [35]. These cells differ from those in other wound types in that they contain high amounts of iron from their engulfment of erythrocytes that have leaked into tissue [35]. In iron-dextran–treated mice, iron laden macrophages isolated from wound margins at day 5 post injury are dominantly pro-inflammatory M1 with an intermediate anti-inflammatory M2 phenotype (TNF-α^hi^, IL-12^hi^, CCR2^hi^, Ly6C^hi^, Dectin-1^med^, IL-4Rα^med^, and CD204^med^) compared with control mice [35]. Treatment of iron loaded mice with the iron chelator desferrioxamine improved wound healing, suggesting that the removal of iron might improve these wounds [35]. Thus, like hypoglycaemia in diabetes, high levels of iron in patients with chronic venous disease leads to an alteration in the macrophage phenotype. Whether these altered macrophages have a defect in efferocytosis, as is seen in diabetes, is yet to be tested. Impaired wound healing is often accompanied by low grade inflammation which can alter cell phenotype, pushing macrophages towards a premature pro-inflammatory phenotype. The high levels of M1 macrophages seen in CVUs results an increased production of TNF which correlates with delayed healing in patients. Addition of recombinant TNF to the margins of an acute wound delays healing [35], while the TNF inhibitors to chronic wounds rescues delayed healing [35,39]. This suggests one of the key factors in wound chronicity is TNF with its increased secretion leading to a delayed phenotype and making TNF a good target to improve wound healing [35].

## 6. Summary

Collectively, these studies suggest that the extended and/or increased presence of M1 macrophages, reduced numbers of M2 macrophages and/or increased M2 activation can all alter the delicate balance of inflammation in the wound and so drastically affect the repair process. Any failure to switch from an M1 phenotype to an M2 phenotype leads to increased inflammation and the secretion of copious amounts of TNF, which inhibits wound closure in chronic wounds. A driver of this M2 switch in wounds is efferocytosis, a process that is dysregulated in diabetic wounds and potentially in CVU, possibly through the chronic inflammation seen in these patients. The anti-inflammatory M2 cells regulate both the repair process and regulate the final scar outcome. These cells secrete growth factors that stimulate proliferation, angiogenesis and fibroblast differentiation to myofibroblasts in the wound. The latter cells secrete collagen, which strengthens the wound but excess collagen leads to increased fibrosis. Great progress has been made in recent years in our understanding of cell types within the wound. Yet there are many more questions to answer, such as how many different phenotypes are present in the wound, what are the key phenotype regulating scar formation and can we improve efferocytosis and the M2 switch in cells from diabetic patients? While macrophage targeted therapies have not been developed, the progress made in the last decade has provided key data that will help inform future research and take us one step further toward perfect healing.

## Figures and Tables

**Table 1:** Human M1 and M2 phenotypes.

Products	M1	M2a	M2b	M2c	M2d	Reference
Marker expression	CD14^++^, CD16^−^, CD68, CD86, CD80, MHC II^high^	CD14^+^ CD16^++^ CD68 CD163 CD206 CD3001, MHC-II^low^	CD14^+^ CD16^++^ CD68 CD86, MHC-II^+^	CD14^+^, CD16^++^, CD68, CD150, CD163 CD206	CD14^+^, CD16^++^, CD68	[9,10,15,16,17]
Cytokines	IL-1β, IL-6, IL-12, IL-18, IL-23, TNF	IL-1ra, sIL-1R, IL-10	IL-1β, IL-6, IL-10, TNF	IL-10	IL-10, IL-12 TNF	[15,18]
Chemokines	CXCL1, CXCL10, CXCL 11, CXCL 16, CCL2-5, CCL8-11	CCL-17, 18, CCL22, CCL24	CCL1 CCL20 CXCL1, CXCL3	CXCL^-^-13, CCL-16, CCL-18 CCR2	CXCL-10, CXCL-16, CCL-5	[19]
Signaling	STAT-1, STAT-4, SOCS-3	STAT-3	SOCS3^+^	STAT-6, SOCS3^+^		[18,20]
Proteases	MMP-1, 2, 3, 7, 9 & 12	MMP-1, 2, 9, 12,13, 14, TIMP-1				[18]
Growth factors/Other	ROS, RNS, NO	EGF, TGFβ, IGF1, Arg1,	COX-2	EGF, TGFβ Arg1,	VEGF, TGFβ iNOS	[10,21]

^−^, negative; ^+^ positive (low); ^++^ positive (high); IL, interleukin; MMP, matrix metalloproteinase; TNF, tumour necrosis factor; MHC, major histocompatibility complex; NOS, Nitric Oxide Synthase; Arg, arginase; ROS, reactive oxygen species; RNS, reactive nitrogen species; EGF, epidermal growth factor; TGFβ, transforming growth factor β; IGF1, Insulin-like growth factor 1; VEGF, vascular endothelial growth factor.

## Abbreviations

AGEs	advanced glycated end products
CVU	chronic venous ulcers
DAMPs	damage associated pattern molecules
ECM	extracellular matrix
IGF-1	insulin-like growth factor-1
PDGFβ	platelet-derived growth factor
TGF-1	transforming growth factor-1
VEGF	vascular endothelial growth factor
cJun-AP1	cJun activator protein 1
IFN-γ	interferon gamma
iNOS	inducible nitric oxide synthase
MMP	matrix metalloproteinase
TIMP	tissue inhibitor of matrix metalloproteinase
TNF	tumour necrosis factor
